# Integrated Diagnostic and Surgical Management of Pediatric CNS Tumors: A Single-Centre Prognostic Analysis

**DOI:** 10.3390/diagnostics16050740

**Published:** 2026-03-02

**Authors:** Peter Spazzapan, Tomaž Velnar

**Affiliations:** Unit of Pediatric Neurosurgery, Department of Neurosurgery, University Medical Centre Ljubljana, Zaloška 7, 1000 Ljubljana, Slovenia; tvelnar@hotmail.com

**Keywords:** pediatric brain tumors, neurosurgery, incidence, cerebellar mutism

## Abstract

**Backgroud/Objectives:** Pediatric central nervous system (CNS) tumors represent the second most common oncological disease in children. The purpose of this research was to analyze the course of surgical treatment for these tumors at the University Medical Centre Ljubljana between October 2018 and December 2025. **Methods:** A retrospective analysis of 110 patients was conducted, focusing on diagnostic accuracy and its correlation with surgical and neurological outcomes. **Results:** Over a seven-year period, 110 children were surgically treated, undergoing a total of 130 operative procedures. A calculated annual incidence of 3.8 cases per 100,000 was identified. The most common initial symptoms were headache (36.3%), vomiting (20%), and ataxia (17.2%). Tumors were localized supratentorially in 52.7% of cases, infratentorially in 30%, and along the spinal canal in 13.6%. The most frequent histopathological types were pilocytic astrocytomas/paediatric-type diffuse low-grade gliomas (18.1%), medulloblastomas (14.5%), and craniopharyngiomas (7.2%). Gross total resection was achieved in 60% of all procedures. Surgical complications occurred in 10.6% of cases, with a surgical mortality rate of 0.9%. Neurological deterioration occurred in 27.2% of cases, most commonly in the form of cerebellar mutism (8.1%). Diagnostic histology of medulloblastoma and infratentorial anatomical location were confirmed as critical prognostic markers for cerebellar mutism (*p* < 0.011) and shunt dependency (*p* = 0.0121). **Conlcusions:** This study confirms that treatment outcomes in the Slovenian tertiary center are comparable to international standards. Integrative diagnostic strategies significantly refine surgical planning and prognostic assessment, optimizing long-term morbidity management.

## 1. Introduction

Pediatric central nervous system (CNS) tumors represent a rare pathology of childhood, although they are the second most common form of pediatric oncological disease [1]. In the literature, the incidence is estimated between 1.15 and 5.14 cases per 100,000 children. Although the histopathological characteristics of CNS tumors in children are similar to those in adults the incidence of various neoplastic forms differs significantly between these two populations [2]. In childhood, astrocytomas are more frequently benign, meningiomas and adenomas are rare, and metastases occur only sporadically [3]. Furthermore, some forms originating from embryonic tissues are common in children and extremely rare in adults [4]. Another essential difference is the anatomical distribution: in adults, the supratentorial location is most common, whereas in children, the supratentorial and infratentorial regions are equally common, each representing approximately half of the cases.

Surgical management must be preceded by an intensive diagnostic phase to assess neurological risks and to define the potential surgical approaches. Furthermore, the prompt identification and diagnosis of surgical complications and neurological deterioration are paramount. Early intervention not only mitigates further injury but also facilitates targeted rehabilitation, ultimately optimizing the patient’s long-term functional recovery.

## 2. Methods

This retrospective study aimed to review the results of surgical treatment for all pediatric CNS tumors treated surgically at the UMC Ljubljana between October 2018 and December 2025. We included patients aged 0 to 17 years who required surgical intervention (biopsy, subtotal, or total resection). We reviewed the children’s demographic data, clinical status, presence of any syndromic diseases, radiological tumor characteristics, surgical methods, and the incidence of surgical and neurological complications. The research was conducted in accordance with the principles of the Declaration of Helsinki; the subjects or their parents agreed to inclusion in the study and freely signed informed consent to participate in the study. The Institutional Ethical Committee approved the retrospective study. Statistical analysis was performed using (SPSS Version 28). Categorical variables were compared using Fisher’s exact test. A *p*-value of <0.05 was considered statistically significant.

## 3. Results

During the study period, 110 children were surgically treated. The median age of patients was 8.6 years; 60 were male and 50 were female. A total of 130 surgical procedures were performed on the population of 110 children: in addition to the primary tumor removal, 8/110 (9%) children required an initial biopsy, 11/110 (10%) required two surgical procedures for tumor removal, and 1/110 (0.9%) case required three operative procedures of tumor resection.

The initial symptoms or clinical issues leading to hospitalization and diagnosis were: headache in 40/110 (36.3%) cases, vomiting in 22/110 (20%), ataxia in 19/110 (17.2%), visual field deficits in 17/110 (15.4%), paresis of one or more limbs in 14/110 (12.7%), and epilepsy in 10/110 (9%). Less common symptoms included cognitive delay, diabetes insipidus, panhypopituitarism, exophthalmos, nystagmus, scoliosis, growth hormone deficiency, sphincter dysfunction, and radiculopathy. Data on preoperative symptoms are collected in Table 1. Ten children had associated syndromic diseases: neurofibromatosis type 1 in 7/110 (6.3%), and one case each of neurofibromatosis type 2, Marfan syndrome, and Lynch syndrome.

The anatomical distribution of tumors was as follows: 58/110 (52.7%) were localized supratentorially, 33/110 (30%) infratentorially, 15/110 (13.6%) along the spinal canal, and 4/110 (3.6%) both supratentorially and infratentorially. More in detail, regions included were: fourth ventricle in 20/110 (18.1%), sella turcica in 11/110 (10%), cerebellar hemispheres in 9/110 (8.1%), frontal lobe in 7/110 (6.3%), thoracic spinal canal in 8/110 (7.2%), and cervical spinal canal in 6/110 (5.4%). The following regions were involved in 5/110 (4.5%) cases each: skull base, supratentorial ventricles, temporal lobe, and third ventricle. Other regions (cerebellopontine angle, optic-hypothalamic region, suprasellar region, pineal region, brainstem, orbit) were involved in fewer than 4 cases each. There were 15 lesions in the spinal canal with the following morphological distribution: 8/15 (53.3%) were extradural, 4/15 (26.6%) were intramedullary, and 3/15 (20%) were intradural extramedullary. Data on tumor localization are collected in Table 2.

Preoperative hydrocephalus required surgical treatment in 16/110 (14.5%) cases. External ventricular drainage was placed in 14 cases, and endoscopic ventriculostomy was successfully performed in 2 cases. Surgical treatment and the choice of approach were conducted according to the characteristics of the lesion, the child, and clinical status. Of the total 130 operations, 8/130 (6.1%) were performed via needle biopsy, 97/130 (74.6%) via microsurgical approaches, and 25/130 (19.2%) via endoscopic approaches. Specific microsurgical approaches included: the telovelar approach for tumors in the fourth ventricle in 32/97 (32.9%), the transcallosal approach for lesions in the lateral and third ventricles in 6/97 (6.1%), the retrosigmoid approach for lesions in the cerebellopontine angle in 3/97 (7.6%), and the pterional and subtemporal approaches for skull base lesions in 6/97 (6.1%) and 2/97 (2%), respectively. Among endoscopic approaches, the endonasal transsphenoidal approach was the most common (14/25, 56%) for lesions in the sellar and suprasellar space. Overall, gross total resection (GTR) was achieved in 78/130 (60%) procedures, subtotal resection in 31/130 (23.8%), while only a biopsy was performed in 21/130 (16.1%). GTR was achieved more frequently in microsurgical approaches compared to endoscopic approaches (*p* < 0.046). Specifically, the data suggests that microsurgical approaches were associated with a higher proportion of GTR (66%) compared to endoscopic approaches (56%), while endoscopic approaches had a higher proportional rate of biopsies (24% vs. 7.2%). Surgical treatment data are collected in Table 3.

The most common histopathological diagnosis was pilocytic astrocytoma/paediatric-type diffuse low-grade glioma in 20/110 (18.1%) cases, followed by medulloblastoma in 16/110 (14.5%) and craniopharyngioma in 8/110 (7.2%). Midline glioma was diagnosed in 5/110 (4.5%) cases, while germinoma, ganglioglioma, anaplastic ependymoma, and glioblastoma were found in 4/110 (3.6%) cases each. Meningioma was present in 3/110 (2.7%) cases, while choroid plexus papilloma, DNET, pineal cyst, and Ewing sarcoma appeared in 2/110 (1.8%) cases each. Other histopathological types occurred in only one case. Histological diagnosis data are collected in Table 3.

Surgical complications occurred in 14/130 (10.6%) cases. These included a subdural hygroma in 4/130 (3%), and diffuse cerebral edema, wound dehiscence, and CSF fistula in 2/130 (1.5%) cases each. Meningitis and epidural hematoma occurred in 1/130 (0.7%) case each. In 8 cases, these complications required surgical intervention. A subduro-peritoneal drainage was placed in 3/130 (2.3%) cases, while external subdural drainage, posterior fossa decompressive craniectomy, wound revision, lumbar drainage, and evacuation of an epidural hematoma were required in 1/130 (0.7%) case each.

Neurological deterioration occurred in 30/110 (27.2%) cases. The most common form was cerebellar mutism, occurring in 9/110 (8.1%) cases (or 27.3% of posterior fossa tumors). There was a significant correlation (*p* < 0.011) between the diagnosis of medulloblastoma and the risk of developing cerebellar mutism. Specifically, 50% of medulloblastoma patients developed CM, compared to only 5.9% of patients with different posterior fossa tumors.

Hemiparesis occurred in 6/110 (5.4%), cranial nerve deficits in 5/110 (4.5%), and diabetes insipidus/panhypopituitarism in 3/110 (2.7%). One case presented with hemianesthesia, ataxia, hyperphagia, and impaired consciousness. Many of these deteriorations were transient; however, permanent neurological deficits persisting months after surgery included: cognitive delay in 8/110 (7.2%), diabetes insipidus and panhypopituitarism in 3/110 (2.7%), hyperphagia in 2/110 (1.8%), epilepsy and ataxia in 1/110 (1.1%). Chronic postoperative hydrocephalus developed in 8/110 (7.2%) cases and was treated surgically with a ventriculo-peritoneal shunt in all instances. A statistically significant correlation was observed between tumor location and the requirement for shunting: children with infratentorial tumors had a higher rate of shunt dependency (18.2%) compared to those with supratentorial lesions (3.4%) (*p* = 0.0121). Furthermore, a comparative analysis of anatomical locations revealed that tumor site was a significant predictor of postoperative neurological morbidity. Patients with infratentorial tumors experienced a significantly higher rate of neurological deterioration compared to those with supratentorial lesions (39.4% vs. 19.0%; Fisher’s exact test, *p* = 0.038). Early postoperative mortality was 0.9%, as one child died due to posterior fossa cerebral edema. Data about surgical complications are collected in Table 4.

Of the 110 children, 11 (9%) required a new surgical procedure for recurrent tumor lesions. In 8/11 (72.2%) cases, the tumor recurred at the same site, and in 3/11 (27.2%) cases at a different site. Of these 11 cases, GTR was achieved in 4 (36.3%), subtotal in 2 (18.1%), and in 1 (9%) case only a biopsy was performed, as the diagnosis of germinoma did not necessitate total removal.

## 4. Discussion

Our study provides a single-centre, retrospective overview of the surgical management of 110 pediatric patients at Slovenia’s national referral centre. While the cohort size is reflective of a smaller national population, the reported surgical outcomes represent a unified technical standard and serve as a critical baseline for assessing the quality of surgical care in Slovenia. Given that, with a few rare exceptions, all pediatric CNS tumors in Slovenia are operated on at UMC Ljubljana, we can consider the incidence at this Institution as the real incidence of this pathology in Slovenia. Based on an average population of 377,000 children and adolescents (0–17 years) in Slovenia during the 2018–2025 period [5], we calculate that the annual incidence was 3.8 per 100,000 children. This is consistent with global literature reporting an incidence between 1.15 and 5.14 per 100,000 [1,2,3,4].

**Figure 1:**
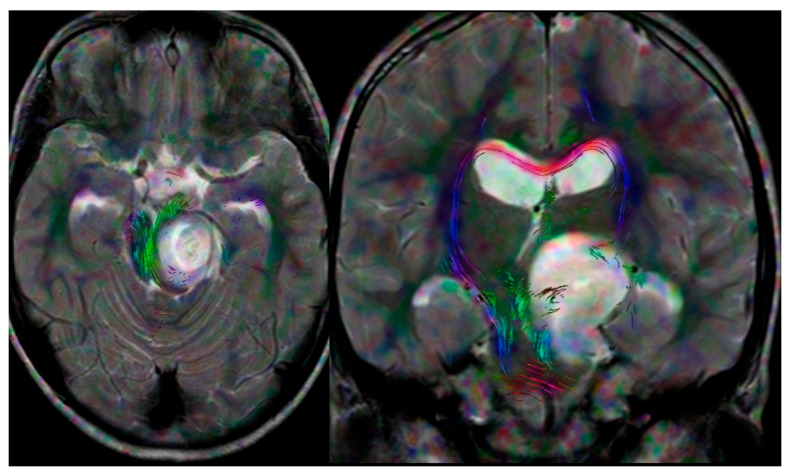
Diffusion tensor imaging (DTI) tractography demonstrating the precise location and orientation of white matter tracts in close proximity to the lesion. In this case, the pyramidal tract is displaced laterally by an extensive brainstem glioma.

The management of pediatric CNS tumors remains one of the most complex challenges in neurosurgery, requiring a delicate balance between aggressive cytoreduction and the preservation of developing neurological function. The surgical treatment of pediatric CNS tumors begins before the operation itself, during the planning phase. It is necessary to assess the child’s neurological and systemic condition. Depending on the anatomical location, specific additional testing is often necessary, like endocrinological testing, audio-vestibular testing and sensorimotor evoked [6]. Beyond CT and MRI, which are crucial for planning, tractography (Figure 1) serves as an additional study to visualize white matter fibers in the immediate vicinity of the tumor [7].

**Figure 2:**
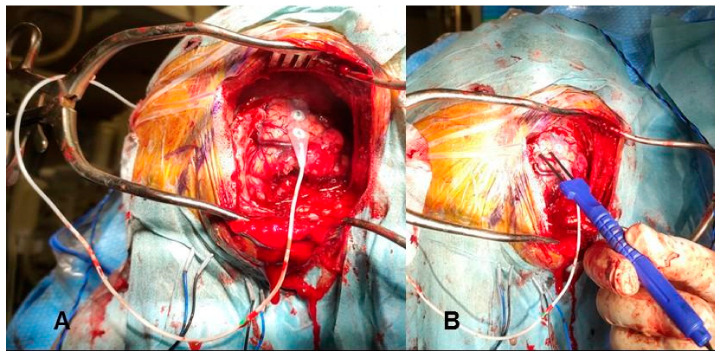
Intraoperative neurophysiological monitoring setup: (**A**) cortical electrodes and (**B**) specialized bipolar stimulating probe utilized for direct cortical and subcortical motor mapping.

**Figure 3:**
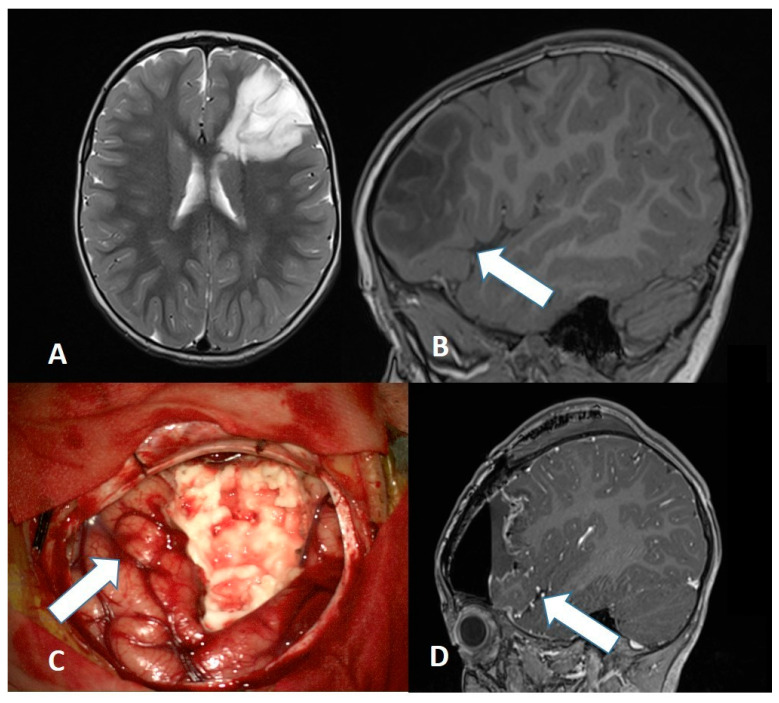
Management of a benign dysembryoplastic neuroepithelial tumor (DNET) (**A**,**B**) involving the Broca area (white arrow). While awake surgery was unfeasible due to the patient’s age, the eloquent speech area was preserved ((**C**), white arrow) using intraoperative neuronavigation, as confirmed by early postoperative MRI (white arrow indicates the anatomically preserved Broca area (**D**)).

Each tumor can usually be accessed via various neurosurgical approaches; the choice depends on tumor characteristics and the surgeon’s experience. The predominance of microsurgical interventions (74.6%) in our study still reflects the continued gold-standard status of open surgery for pediatric tumors [8,9]. Microsurgical techniques and approaches can be aided by technological support, particularly by neuronavigation (Figure 2) and neuromonitoring/mapping (Figure 3), which are of great help to localize eloquent regions [6,10].

**Figure 4:**
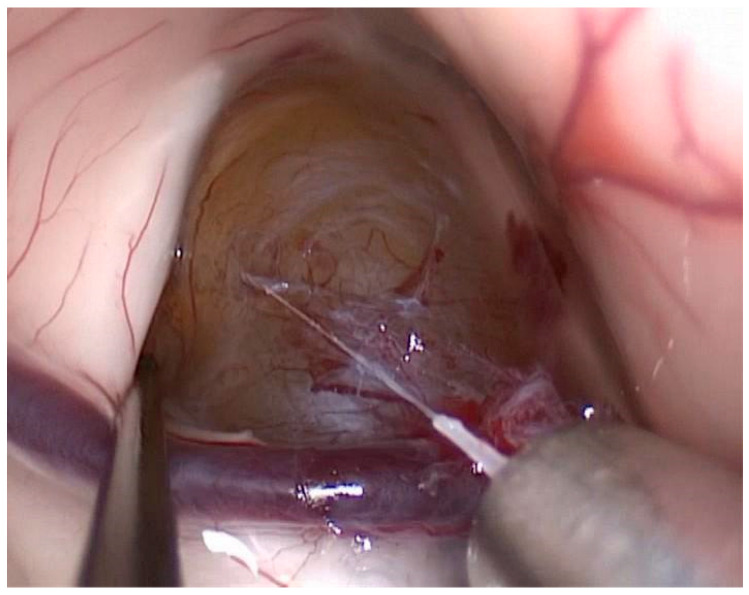
Purely endoscopic resection of a colloid cyst located at the roof of the third ventricle.

Our use of endoscopy (19.2% overall), particularly of the transsphenoidal approach for 56% of endoscopic cases, aligns with the overall growing indications for endoscopic transsphenoidal surgery in the pediatric population, primarily for craniopharyngiomas and pituitary lesions. This approach has been shown to offer superior visualization and reduced hospital stays compared to transcranial routes [11,12]. Purely endoscopic procedures (Figure 4) are rarely used for CNS tumor removal due to limited hemorrhage control; instead, endoscopically assisted microsurgery is more common.

In our study, the microsurgical techniques achieved a higher proportion of GTR (66%), compared to endoscopic approaches, which were more frequently utilized for biopsies (24%). The statistical difference in GTR rates between microsurgery and endoscopy (*p* = 0.046) reflects a diagnostic selection bias where endoscopy is prioritized for tissue diagnosis in high-risk midline regions, whereas microsurgery remains the mainstay for achieving total tumor removal in accessible compartments. Overall, we achieved GTR in 60% of cases. This result is competitive with those reported by international benchmarks where GTR rates typically range between 55% and 75% [13].

### 4.1. Strategies for Different Histopathological Types of Tumor

The histopathological distribution in our series is spearheaded by pilocytic astrocytomas/paediatric-type diffuse low-grade gliomas (18.1%) and medulloblastomas (14.5%), followed by craniopharyngiomas (7.2%). These findings are broadly consistent with larger studies, which identify low-grade gliomas and embryonal tumors as the most prevalent pediatric CNS tumors [1,2,3,4,14]. Similarly, our distribution of malignant versus benign lesions remains comparable, suggesting no significant referral bias in our national system [15].

**Figure 5:**
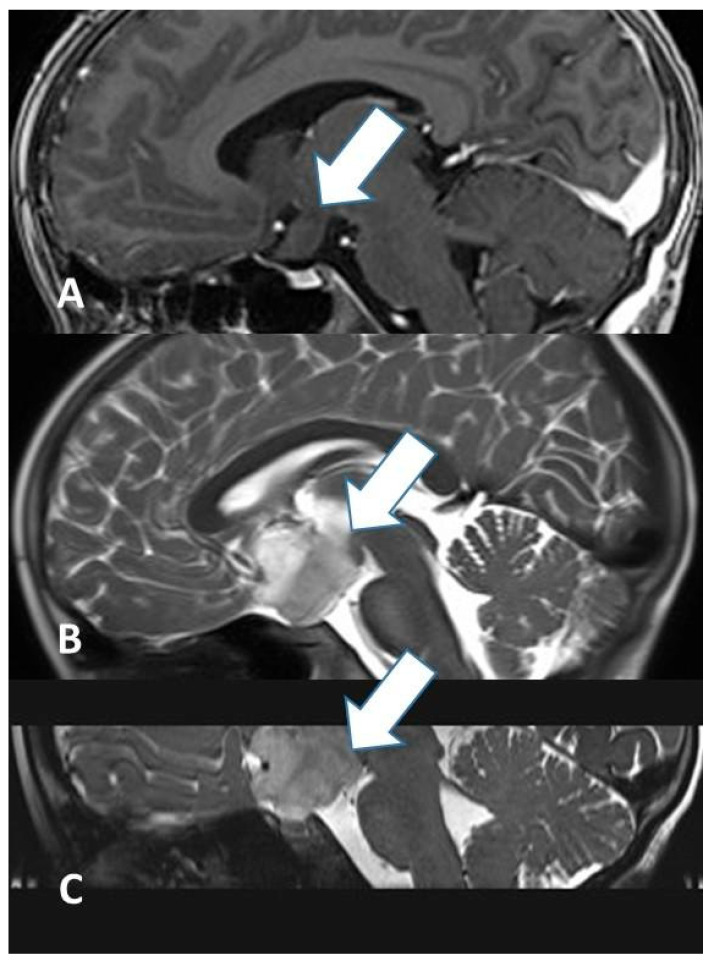
Longitudinal MRI surveillance of an optic pathway glioma in a patient with Neurofibromatosis type 1. Images (**A**–**C**) show the progressive growth of the optic glioma, indicated by the white arrows. A multidisciplinary strategy was implemented; to avoid permanent visual morbidity associated with radical resection, the patient underwent biopsy followed by chemotherapy.

**Figure 6:**
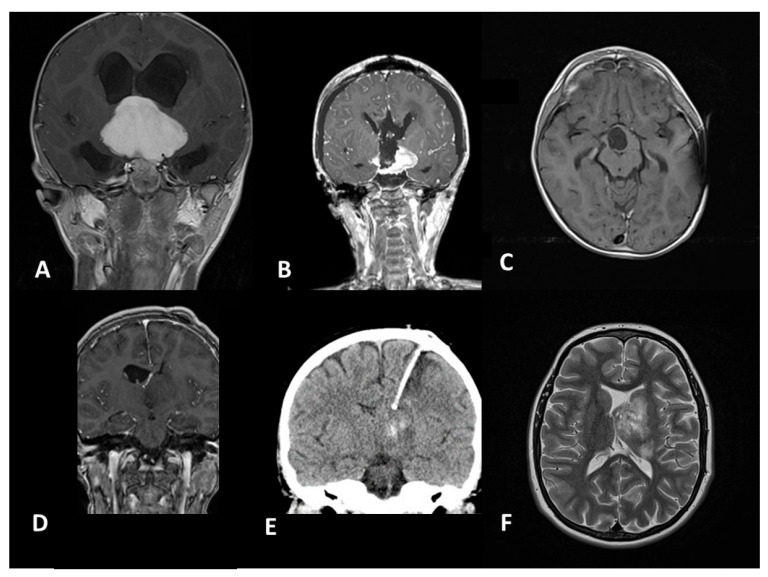
Management of pilocytic astrocytomas/paediatric-type diffuse low-grade gliomas in eloquent regions. Case 1 (**A**–**C**): Following subtotal resection (**B**), adjuvant chemotherapy resulted in complete radiological resolution (**C**). Case 2 (**D**–**F**): A thalamic pilocytic astrocytoma/paediatric-type diffuse low-grade glioma presenting with hydrocephalus treated via ventriculoperitoneal shunt (**E**). After endoscopic biopsy, chemotherapy ensured tumor stability (**F**) with preservation of neurological function.

Different histopathological types vary in their sensitivity to oncological treatment [16]; therefore, surgical radicality must be adapted to different lesions. When a tumor is localized deep in the brain and its removal carries a high risk of neurological damage, an initial needle biopsy is warranted. Histological diagnosis then allows for the best multidisciplinary strategy. For pilocytic astrocytomas/paediatric-type diffuse low-grade gliomas, the goal is radical resection, as this can offer a complete cure. If growth extends into eloquent regions despite its benign nature, subtotal resection followed by chemotherapy is appropriate. This particularly applies to optic gliomas in Neurofibromatosis type 1 (Figure 5), optic-hypothalamic gliomas (Figure 6), and brainstem or spinal astrocytomas, where total removal would cause irreversible deficits [16,17]. For unresectable low-grade gliomas, five-year progression-free survival rates of nearly 50% can be achieved with chemotherapy, emphasizing that surgical subtotal resections in eloquent areas do not preclude long-term stability [18].

**Figure 7:**
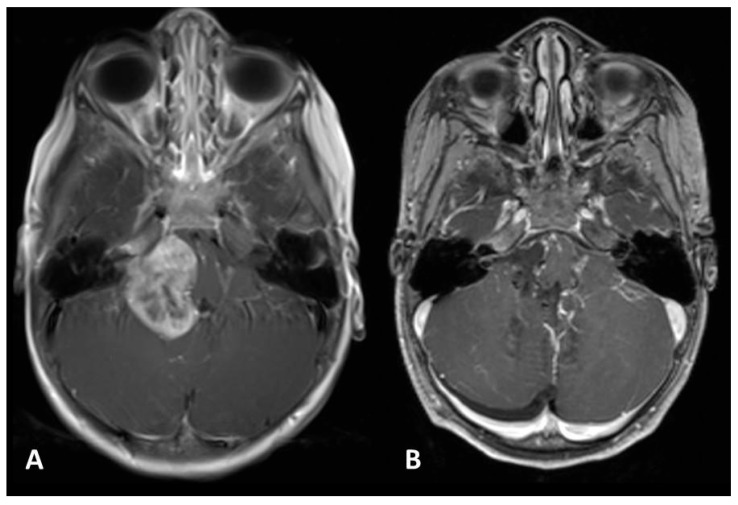
Infratentorial ependymoma (**A**) extending from the cerebellopontine angle through the Luschka foramen into the fourth ventricle. Radical surgical excision (**B**) remains the primary treatment modality but is technically challenging due to the proximity of critical brainstem structures.

Conversely, for germinomas, a biopsy suffices, as they are sensitive to chemotherapy [19]. Large multi-institutional series have confirmed that for germinomas, biopsy followed by adjuvant therapy yields cure rates exceeding 90% [20]. The opposite is true for ependymomas, where the most complete surgical removal possible must be achieved (Figure 7). These tumors respond poorly to oncology treatments and tend to recur. Data from the Ependymoma Outcomes Group show that the 5-year overall survival drops significantly from 70–80% with GTR to below 30–40% when only subtotal resection is achieved, reinforcing our commitment to aggressive resection in these cases [21].

**Figure 8:**
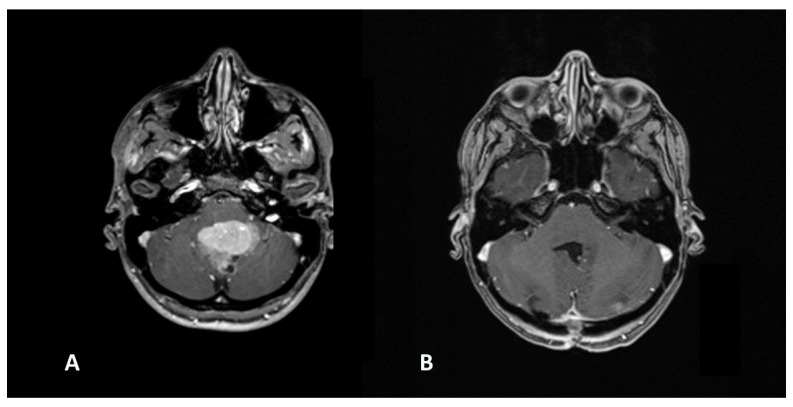
Medulloblastoma occupying the fourth ventricle (**A**). GTR (**B**) was achieved via a telovelar approach, minimizing vermal disruption.

Medulloblastomas are embryonic tumors of the fourth ventricle. Prognosis depends on genetic features, and treatment combines surgery, chemotherapy, and radiotherapy. Recent molecular subgrouping suggests that while GTR remains a prognostic pillar (Figure 8), the presence of <1.5 cm^3^ of residual tumor in some molecular subtypes (WNT-subgroup) may carry a similarly excellent prognosis, allowing for slightly more conservative margins in specific molecular contexts to avoid the occurrence of cerebellar mutism [22,23].

Craniopharyngiomas pose a challenge due to the proximity of the hypothalamus. Surgical damage here leads to severe hormonal/electrolyte imbalances, diabetes insipidus, loss of hunger regulation, hyperphagia, and obesity. Modern treatment involves surgery with maximal hypothalamic preservation, postoperative chemotherapy, and targeted proton radiation [24]. Patients with limited resection and radiotherapy have been reported to have significantly better quality-of-life scores and lower rates of morbid obesity compared to those who underwent radical hypothalamic dissection [25].

**Figure 9:**
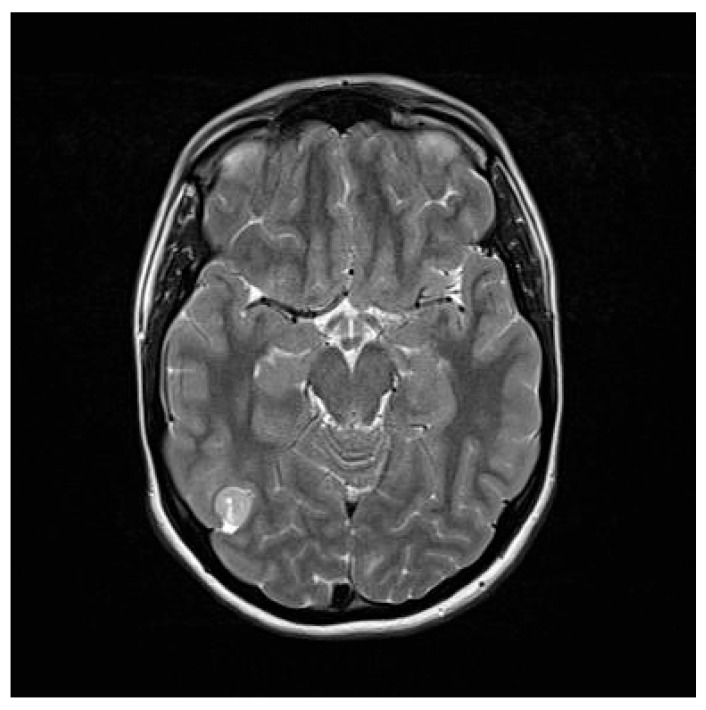
Benign ganglioglioma presenting with refractory epilepsy. Complete surgical excision is the definitive treatment to achieve seizure freedom.

For epilepsy-associated tumors (DNET, gangliogliomas), resection usually cures the epilepsy (Figure 9), but the procedure must be extended to all areas from which epileptic activity originates, requiring precise preoperative EEG localization. In a large meta-analysis, seizure freedom was achieved in 75–80% of cases following early surgical intervention, with superior outcomes linked to shorter durations of preoperative epilepsy [26].

### 4.2. Complications

Surgical complications in complex neurosurgery can be frequent; meticulous planning and technique are required to minimize them. Precise dural suturing and careful compressive bandaging after posterior fossa surgery can significantly reduce the risk of wound dehiscence, CSF leaks, and subsequent meningitis. Conversely, surgical technique plays a significantly smaller role in preventing cerebellar mutism. In the literature, its incidence in cerebellar surgery ranges from 5% to 35%, appearing mostly in small children with large fourth ventricle lesions, particularly medulloblastomas [27,28,29]. While traditional views blamed vermal damage, mutism also occurs in the telovelar approach if compression, ischemia, or edema occurs near the dentate nuclei [29]. It often appears with a 24–48-h delay as swelling peaks. It is almost never permanent in its most severe form, suggesting the brain adapts as edema subsides, though recovery is often accompanied by dysarthria. While cerebellar mutism is a known risk in posterior fossa surgery, our data confirms the histopathological diagnosis as a critical risk-stratifier. We found a highly significant correlation between medulloblastoma and cerebellar mutism and this confirms that the biological behaviour of medulloblastoma creates a high-risk surgical environment that surpasses the risk posed by the anatomical approach alone.

Overall, children undergoing surgery for infratentorial tumors had 2.77 times higher odds of experiencing a postoperative neurological deficit compared to those with supratentorial tumors. While supratentorial tumors were more frequent in your cohort (52.7%), the surgical burden in terms of morbidity was disproportionately concentrated in the posterior fossa. It is worth noting that the tumors spanning both supra and infratentorial compartments showed a very high rate of deterioration (75%). While the sample size is too small (n = 4) for a robust independent test, it suggests that this multicompartmental involvement is an even higher risk factor.

Preoperative hydrocephalus requiring surgical intervention was present in 14.5% of our cohort. The heavy reliance on external ventricular drainage suggests a strategy focused on immediate intracranial pressure control. While some centers advocate for routine preoperative endoscopic third ventriculostomy to avoid shunting, our results suggest a more tailored approach, which emphasises that not all cases evolve into a chronic hydrocephalus. This chronic complication occurred in 7.2% in our series. Literature suggests hydrocephalus persists in 10% to 40% of patients after tumor resection [30]. The incidence is particularly high in posterior fossa tumors, especially in medulloblastomas, where the need for permanent drainage can rise to 50%. Ventriculoperitoneal shunting remains the gold standard, preventing secondary damage to the developing brain. Risks for chronic hydrocephalus are linked to age lower than 3 years, midline location, and extent of resection [30]. The development of chronic postoperative hydrocephalus in our series (7.2%) was not random but strictly linked to tumor location. The statistically significant correlation (*p* = 0.0121) between infratentorial tumors and the need for a ventriculoperitoneal shunt underscores the high risk of a permanent disruption of CSF dynamics after posterior fossa surgery, mainly due to scarring or functional impairment of the aqueduct and fourth ventricle outlets.

## 5. Conclusions

This seven-year retrospective analysis reflects the incidence of pediatric tumors within Slovenia’s population of two million and demonstrates the specific surgical outcomes and complications. The results confirm that the neurosurgical management of pediatric CNS tumors in Slovenia achieves a high level of safety and efficacy, comparable to established international centers. The annual incidence (3.8 per 100,000 children) and predominant histopathological types reflect global trends. Results highlight the key role of preoperative planning and modern technologies (neuronavigation, endoscopic-assisted microneurosurgery) in enabling high rates of total resection (60%) with low perioperative mortality (0.9%). Despite technical progress, cerebellar mutism remains a major challenge, affecting nearly a third of posterior fossa tumor cases.

## Figures and Tables

**Table 1:** Preoperative signs and symptoms among the 110 children included in the study.

Clinical Presentation	Number of Cases (n)	Percentage (%)
Headache	40	36.3%
Vomiting	22	20.0%
Ataxia	19	17.2%
Visual Field Deficits	17	15.4%
Paresis (one or more limbs)	14	12.7%
Epilepsy	10	9.0%
Cognitive Delay	8	7.2%
Diabetes Insipidus	3	2.7%
Panhypopituitarism	3	2.7%
Hyperphagia	2	1.8%
Other	12	10.9%
Total Patients	110	100%

**Table 2:** Location of the tumors in the CNS.

Location	Number of Cases (n)	Percentage (%)
Supratentorial	58	52.7%
Sellar/Suprasellar region	11	10.0%
Frontal lobe	7	6.3%
Ventricles (Lateral/Third)	10	9.0%
Temporal lobe	5	4.5%
Other	25	22.7%
Infratentorial	33	30.0%
Fourth Ventricle	20	18.1%
Cerebellar Hemispheres	9	8.1%
Cerebellopontine Angle	4	3.6%
Supratentorial and infratentorial	4	3.6%
Spinal segment	15	13.6%
Thoracic	8	7.2%
Cervical	6	5.4%
Lumbar	1	0.9%
Spinal localization		
Extradural	7	46.6%
Intramedullary	4	26.6%
Intradural Extramedullary	3	20.0%
Total	110	100%

**Table 3:** Surgical treatment, surgical approaches and histopathological diagnoses of the 110 children included in the study.

Category	Sub-Category	n/Total	Percentage (%)
Preoperative Hydrocephalus	Total requiring treatment	16/110	14.5%
	External Ventricular Drainage	14/16	87.5%
	Endoscopic Ventriculostomy	2/16	12.5%
Surgical Approach	Microsurgical	97/130	74.6%
	Telovelar	32/97	32.9%
	Retrosigmoid	3/97	3.1%
	Transcallosal	6/97	6.1%
	Pterional	6/97	6.1%
	Subtemporal	2/97	2.0%
	Endoscopic	25/130	19.2%
	Transsphenoidal	14/25	56.0%
	Needle Biopsy	8/130	6.1%
Extent of Resection	GTR	78/130	60.0%
	Subtotal Resection	31/130	23.8%
	Biopsy	21/130	16.1%
Histopathological Diagnosis	Pilocytic Astrocytoma/paediatric-type diffuse low-grade glioma	20/110	18.1%
	Medulloblastoma	16/110	14.5%
	Craniopharyngioma	8/110	7.2%
	Midline Glioma	5/110	4.5%
	Germinoma	4/110	3.6%
	Ganglioglioma	4/110	3.6%
	Anaplastic Ependymoma	4/110	3.6%
	Glioblastoma	4/110	3.6%
	Meningioma	3/110	2.7%
	Choroid Plexus Papilloma	2/110	1.8%
	DNET	2/110	1.8%
	Pineal Cyst	2/110	1.8%
	Ewing Sarcoma	2/110	1.8%
	Others	34/110	30.9%
	Total	110/110	100%

**Table 4:** Surgical complications among the 110 children included in the study.

Category	Outcome	n/Total	Percentage (%)
Surgical Complications		14/130	10.6%
	Subdural hygroma	4/130	3.0%
	Diffuse cerebral edema	2/130	1.5%
	Wound dehiscence	2/130	1.5%
	CSF fistula	2/130	1.5%
	Meningitis	1/130	0.7%
	Epidural hematoma	1/130	0.7%
Surgical Revisions		8/130	6.2%
	Subduro-peritoneal shunt	3/130	2.3%
	Evacuation of hematoma	5/130	3.8%
Neurological Outcomes		30/110	27.2%
	Cerebellar mutism	9/110	8.1%
	In Posterior Fossa tumors	9/33	27.3%
	Hemiparesis	6/110	5.4%
	Cranial nerve deficits	5/110	4.5%
	Diabetes insipidus/Panhypopituitarism	3/110	2.7%
	Cognitive delay	8/110	7.2%
Ventriculoperitoneal Shunt		8/110	7.2%
Early postoperative mortality		1/110	0.9%

## Data Availability

The data presented in this study are available upon request from the corresponding author.
